# The influence of the pavement friction coefficient evolution caused by traffic flow on the risk of motorway horizontal curves

**DOI:** 10.1371/journal.pone.0266519

**Published:** 2022-08-22

**Authors:** Guilong Xu, Jinliang Xu, Huagang Shan, Chao Gao, Jinsong Ran, Yongji Ma, Yuhong Yao

**Affiliations:** 1 School of Highway, Chang’an University, Xi’an, China; 2 Shaoxing Transportation Investment Group Co., Ltd., Shaoxing, Zhejiang, China; Tongji University, CHINA

## Abstract

The friction coefficient between the tire and the road is one of the key parameters affecting road traffic safety. The purpose of this paper is to quantify the risk of skidding for the vehicles due to the friction evolution caused by the traffic polishing in the horizontal curve. Based on the reliability theory, an innovative dynamic risk assessment model is developed in the present study for passenger cars and trucks. The influence of two traffic characteristics for pavement friction is quantified: cumulative traffic volume (CTV) and annual average daily traffic of trucks (AADTT). The speed distribution on the horizontal curve of the motorway is obtained through field experiments as the basic parameter of the model. The Hasofer-Lind Method is adopted to solve the reliability and the risk probability of vehicle skidding. The results show that in the traffic characteristics, the AADTT has a significant impact on the pavement friction; When the AADTT on the road exceeds 2000 veh/d, the increasing CTV leads to friction decrease rapidly and therefore has a significant impact on the risk of horizontal curve. Especially for roads with more than 50 million vehicles of the CTV, the risk of the horizontal curve shows a sharp increase with CTV rising. The research results can provide reference for the road maintenance department to determine the timing of road maintenance.

## 1. Introduction

The friction coefficient between the tire and the road seriously affects the traffic safety. Especially in the horizontal curve, insufficient friction coefficient will significantly increase the severity of traffic accidents. According to the National Highway Traffic Safety Administration (NHTSA), approximately 6.4 million people in the United States involves in traffic accidents each year, 3 million people are injured in the accident, and 42,000 people die. Among these accidents, fatal accidents related to wet roads accounted for 13.5%, and traffic accidents accounted for 25% [1]. Besides, according to the accident data and road performance data provided by the Texas Department of Transportation (TxDOT), Buddhavarapu, Banerjee [2] analyzed the traffic accidents that occurred on the horizontal curve from 2006 to 2009. They found that there was a significant negative correlation between the lateral friction coefficient on the horizontal curve and the severity of the accident. In other words, poor tire-pavement friction coefficient would lead to more severe accidents.

Friction coefficient is mainly depended on the road surface texture. which is defined by two scale levels: micro-texture and macro-texture [3]. These two textures varies under the polishing of cumulative traffic volume (CTV) [4], which leads to the reduction of friction coefficient during road life [5, 6]. Some studies have analyzed the evolution of friction coefficient caused by traffic polishing. Do, Tang [7] used a polisher to continuously polish the produced samples in the laboratory, and measured the roughness parameter changes of the aggregate surface to characterize the evolution of the friction coefficient under the traffic polishing. After some scholars noticed the evolution of friction coefficient under the traffic polishing, they began to quantify the influence model of traffic on road friction coefficient in the laboratory. By polishing the samples in the laboratory, Kane, Zhao [8] analyzed the influence of aggregate type, traffic level and vehicle speed on the friction coefficient, and established an evolution model of the friction coefficient. Hofko, Kugler [5] simulated the traffic polishing in the laboratory, and proposed a prediction model of friction coefficient under different traffic levels. These studies show that considering the importance of friction coefficient for traffic safety [9], its evolution under CTV has attracted widespread attentions.

In light of the above results, it can be found that long-term polishing effects of CTV will lead to pavement friction deterioration. This deterioration may result in potential skidding risk for the vehicles on the horizontal curve [10, 11]. Because when the vehicles traverse a horizontal curve, the friction supply from pavement plays a critical role against the centrifugal force to stay driving stability of vehicles [12]. If the friction coefficient supply from the road can’t meet the friction demand for turning, the vehicles will involve in departure [1, 13]. Therefore, it is significantly worthy to investigate the influence of the friction evolution for traffic safety. However, as far as we know, limited efforts have been undertaken to quantify this risk from the perspective of friction coefficient evolution caused by CTV.

Therefore, this paper aims to investigate the influence of the friction evolution due to traffic polishing on the traffic safety. Based on the reliability theory, this paper proposes a dynamic risk assessment model from a novel perspective of the friction evolution under traffic polishing. Probability of failure (POF) and reliability are employed to assess the skidding risk for passenger cars and trcuks. The research results can evaluate the safety of existing roads in different stages, and provide a reference for road maintenance departments to determine the timing of road maintenance.

The remaining parts of this article are arranged as follows: Section 2 introduces the reliability theory and the risk assessment model is developed. In section 3, the reliability and probability of failure are adopted to quantify the impact of the friction coefficient evolution caused by the traffic for the skidding risk. And some suggestions for ensure traffic safety are put forward. Section 4 summarizes the main research conclusions obtained in this paper.

## 2. Method

Based on the reliability theory, this paper utilizes the road friction evolution model proposed by Hofko, Kugler [5] to construct the friction coefficient supply. The friction coefficient demand for the vehicle to safely traverse the curve is calculated by the dynamic model. Finally, a risk assessment model is proposed based on the supply and demand of friction coefficient.

Among the basic parameters of the above model, the speed of the vehicle and the mean profile depth (MPD) are considered as random variables. The speed data of the vehicle on the horizontal curve was collected by the laser gun. K-S test was adopted to verify that the speed of vehicles has normal distribution. MPD data refers the results collected by Plati and Pomoni [14]. The radius of the horizontal curve, the superelevation, the height of the center of gravity of the vehicle, and the height of the center of rotation of the suspension system are considered as deterministic variables. The radius and superelevation of the horizontal curve adopt the data of Xianyang-Chunhua motorway in Shaanxi Province, China, supplied by CCCC First Highway Consultants Co.Ltd. The gravity center height of the vehicle and the rotation center height of the suspension system refer the values recommended by Gillespie [15] in 《Fundamentals of vehicle dynamics》.

**Fig 1:**
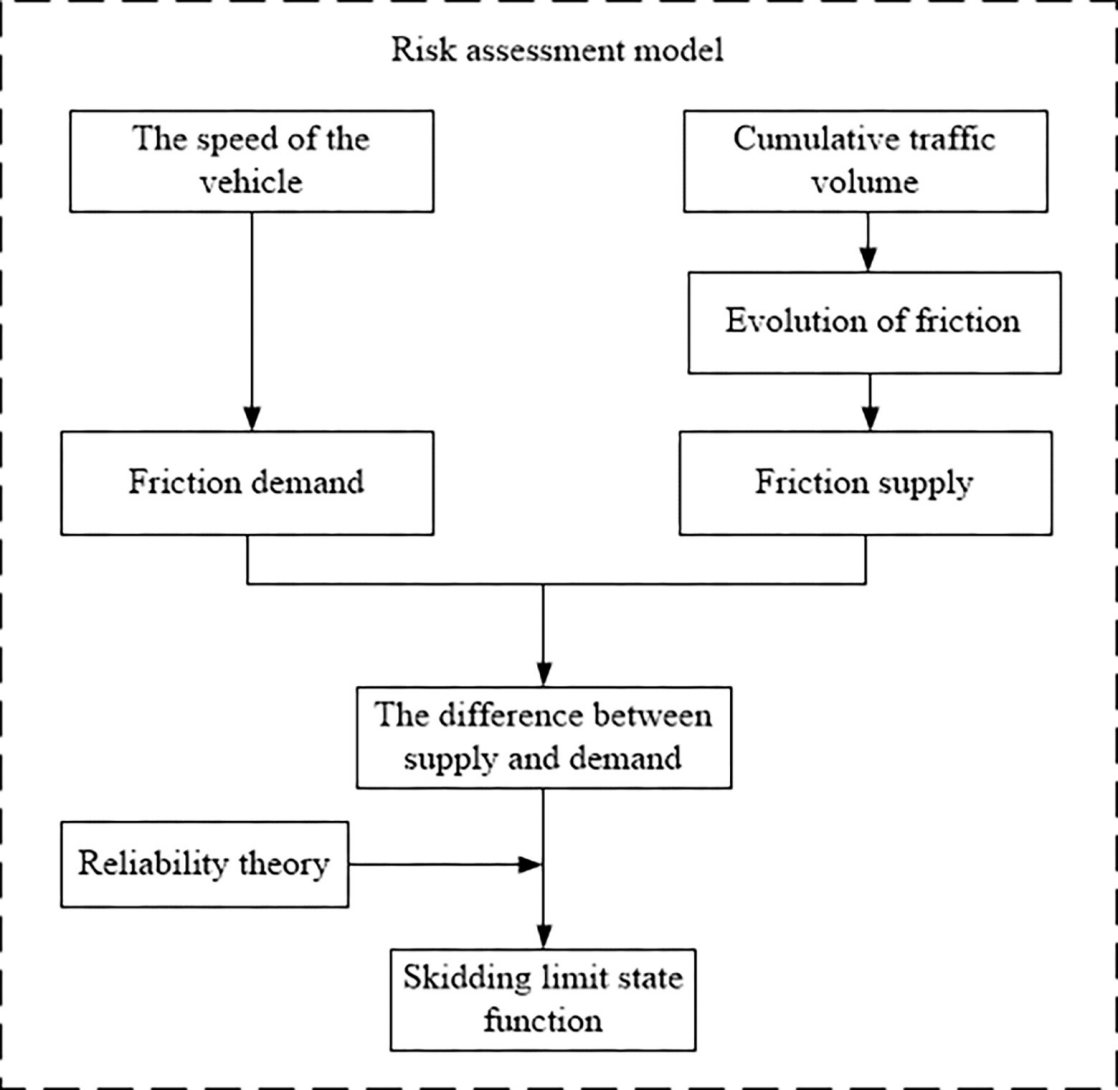
Method flow chart.

The flowchart of the above method is shown in Fig 1, and the specific process is described in detail as follows.

## 2.1 Reliability theory

In the engineering structure, assuming that the load bearing capacity of the structure is *Re* and the load is *S*, the performance function *Z* of the structure is defined as Eq (1):

Z=Re−S (1)

The distribution of structural performance function *Z* can be obtained by using the distribution of structural resistance *Re* and load *S*. Reliability $\beta =\frac{\mu_{Z}}{\sigma_{Z}}$. The probability of failure expression *P*_*f*_ can be given by Eq (2):

$$P_{f}=P(Re<S)=P(Z<0)=1-\Phi (\beta )$$ (2)

When the structural performance function is determined by multiple variables, it can be written as *Z* = g(*X*) = *g*(*X*_1_,*X*_2_,…,*X*_*n*_). *X*_*i*_(*i* = 1,2,…,*n*) represents the response variable of the structural performance function. The limit state of the structure is expressed as g(*X*) = 0. When the structural performance function Z = g(*X*) is a linear or non-linear expression of the response variable, Hasofer and Lind [16] proposed the Hasofer-Lind Method (HLM) to solve the structural probability of failure *P*_*f*_.

In the model established in this paper, the friction supply from the road surface represents the structural resistance *Re* and the demand for automobile anti-skid represents the load *S*. The random factors affecting the supply and demand of the friction are adopted as the response variable *X*_*i*_. The structural performance function *Z* is expressed by the difference between the supply and demand of the friction.

### 2.2 Risk assessment model

#### 2.2.1 Evolution of friction supply

Under long-term polishing effects of traffic flow, the friction coefficient of the road surface will decrease. Hofko, Kugler [5] used the cumulative traffic volume(CTV) and the annual average daily traffic of trucks (AADTT) to developed the friction evolution model of the asphalt pavement, which is expressed by Eq (3):

$$f_{60}=-0.039\ln(PP)+0.7357$$ (3)

Where: *f*_60_- the road friction coefficient when the vehicle speed is 60km/h. *PP*- the number of polishing times of traffic flow, given by Eq (4):

$$PP=5336.6\cdot CTV_{W,AADTT}-5099.5$$ (4)

Where: *CTV*_*W*,*AADTT*_ - the weight of traffic flow, calculated by Eq (5):

$$CTV_{W,AADTT}=\frac{CTV\cdot AADTT}{10^{6}\cdot 10^{4}}$$ (5)

Where: *CTV*-cumulative traffic volume (veh), *AADTT*- annual average daily traffic of trucks (veh/d).

The friction coefficient *f*_60_ under the different traffic polishing can be calculated by Eqs (3)–(5), but this model is only applicable for a vehicle with a speed of 60km/h. In light of this, a converted model proposed by Wambold, Antle [17] is employed to determine the friction coefficient at different driving speeds, which is shown in Eq (6).

$$f_{V}=f_{60}\cdot \exp(\frac{60-V}{S_{p}})$$ (6)

Where: *f*_*V*_- The friction coefficient when the car speed is *V*. *V*- Driving speed of car (km/h). *S*_*p*_ is the speed constant (km/h) and its value is related to the pavement texture structure. The equation is expressed as below:

$$S_{p}=a+b\cdot T_{X}$$ (7)

Where: a, b- the constants of texture structure measuring equipment. The values of reference Donnell, Wood [18]: *a* = 14.32, *b* = 89.7. *T*_*X*_- mean profile depth (MPD), value from 0.5 to 50mm [19].

It should be noted that Hofko, Kugler [5] measures the coefficient of friction in the straight. When the car is traversing a curve, the radial friction coefficient is 0.925 times that of the straight [20]. The side friction coefficient supplied by the road surface can be obtained by Eq (8):

$$f_{supply}=0.925f_{V}$$ (8)

Where: *f*_*supply*_- the friction coefficient supplied from the pavement. As for trucks, studies have shown that the side friction coefficient of the truck is only 70% of the car’s [21].

#### 2.2.2 Skidding limit state function

It is important to select the appropriate vehicle models for the safety assessment. The general vehicle models include pint-mass model, suspended vehicle model, bicycle model, multi-body simulation model [22]. A more complex model means that a larger number of parameters are needed with higher order differential equations. The pint-mass model and suspended vehicle model were selected for the simplicity in this paper.

**Fig 2:**
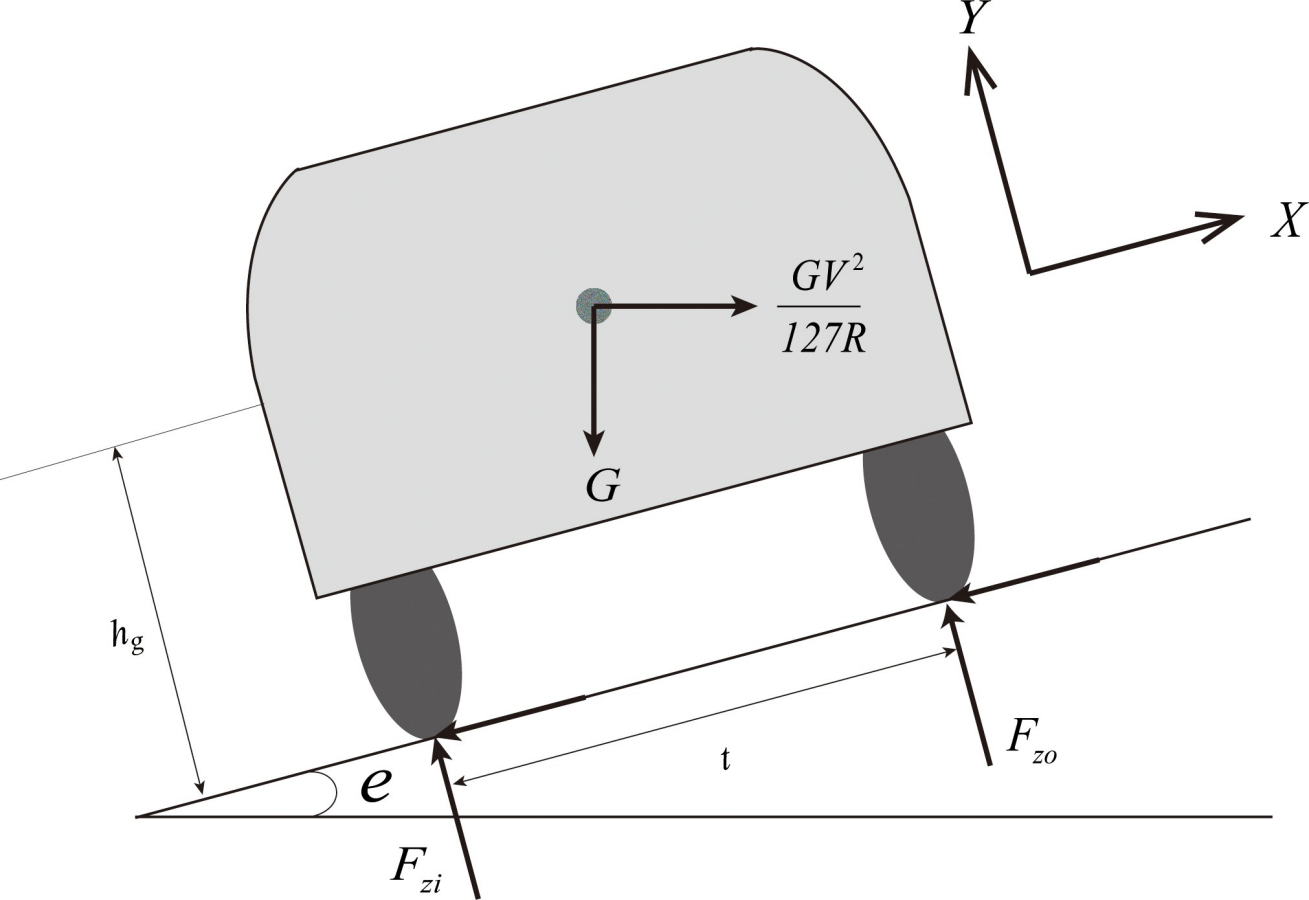
Point-mass model.

The current road design theory [12, 23] regards the vehicle as a point-mass model, which is shown in Fig 2. The radius of the horizontal curve is calculated by Eq (9):

$$R=\frac{v^{2}}{g(f+e)}=\frac{V^{2}}{127(f+e)}$$ (9)

Where: *R*- Horizontal curve radius, *g*- Gravitational acceleration (9.81m/s^2^), *v*- Driving speed (m/s), *f*- Side fraction factor, *e*- Superelevation. The anti-skid friction demand *f*_*p−demand*_ of the mass point model is shown in Eq (10):

$$f_{p-demand}=\frac{V^{2}}{127R}-e$$ (10)

**Fig 3:**
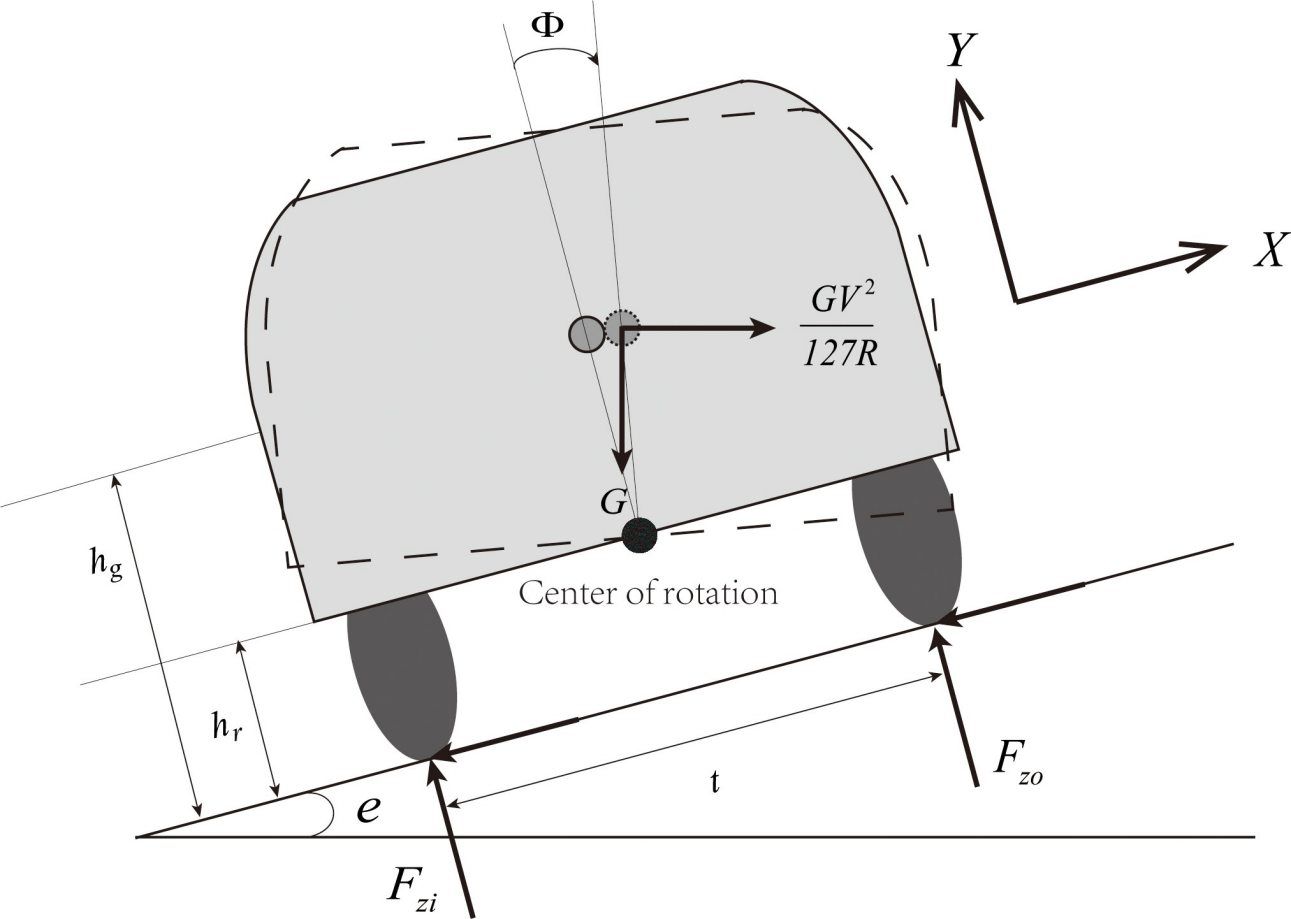
Vehicle model with suspension system.

However, the suspension system has been equipped with almost all vehicles. When the vehicle traverses a horizontal curve, the suspension system will rotate outward under the centrifugal force. The force diagram is shown in Fig 3. In order to improve the defects of the point-mass model, Chang [24] analyzed the mechanical stability of a vehicle with a suspension system when driving in a curve, and derives radius based on the vehicle’s sideslip stability, which is given by:

$$R=\frac{V^{2}}{127[(1-\frac{h_{r}}{h_{g}})e+f]}[1+r_{\Phi }(1-\frac{h_{r}}{h_{g}})]$$ (11)

Where: *h*_*r*_- the distance from the center of rotation to the road surface (m). *h*_*g*_- the distance from the center of gravity of the vehicle to the road surface (m). *r*_Φ_- the rotation rate of the suspension system. From Eq (11), the anti-skid friction demand *f*_*s−demand*_ of a vehicle with a suspension system can be obtained as:

$$f_{s-demand}=\frac{V^{2}}{127R}[1+r_{\Phi }(1-\frac{h_{r}}{h_{g}})]-e(1-\frac{h_{r}}{h_{g}})$$ (12)

The skidding limit state function *Z*_1_ of the mass-point model can be obtained by Eq (10), which is shown as:

$$Z_{1}=f_{supply}-f_{p-demand}=f_{supply}-\frac{V^{2}}{127R}+e$$ (13)

From Eq (12), the skidding limit state function *Z*_2_ of a vehicle with a suspension system is obtained as:

$$\begin{array}{l} Z_{2}=f_{supply}-f_{s-demand}\\ \;=f_{supply}-\frac{V^{2}}{127R}\left[1+r_{\Phi }(1-\frac{h_{r}}{h_{g}})\right]+e\left(1-\frac{h_{r}}{h_{g}}\right) \end{array}$$ (14)

### 2.3 Data collection

The radius, superelevation, height of the center of gravity and the rotation center of the vehicle are regarded as deterministic variables. The MPD and the speed are regarded as random variables. Specific data information is described below subsections.

#### 2.3.1 Speed

The geometric characteristics of the road significantly affect the speed selection behavior of the driver [25]. Some studies have explored the speed distribution in the horizontal curve and predicted that based on the road geometric characteristics (e.g. radius, longitudinal gradient) [26, 27]. Sil, Nama [28] and Himes [29] verified that the speed of a car on a horizontal curve has a normal.

This study collected vehicle speed data in a free flow state for skidding risk assessment. The Xianyang-Chunhua Motorway in Shaanxi Province, China with a small traffic volume (250 veh/h in the peak hour) is convenient for collecting vehicle speeds under free flow conditions, so it was a good choice for experimentation. The midpoint of a horizontal curve with a radius of 1000m is selected as the speed measurement point. There is no speed limit sign on the experimental road section with a good sight distance. The geometric data of the field road is provided by CCCC First Highway Consultants Co.Ltd, as shown in Table 1.

**Table 1:** Geometric data.

Radius (m)	Design speed (km/h)	Posted speed (km/h)	Lane	Length (m)	Gradient (%)	Superelevation (%)
1000	100	100	4	574	2.84	5

**Fig 4:**
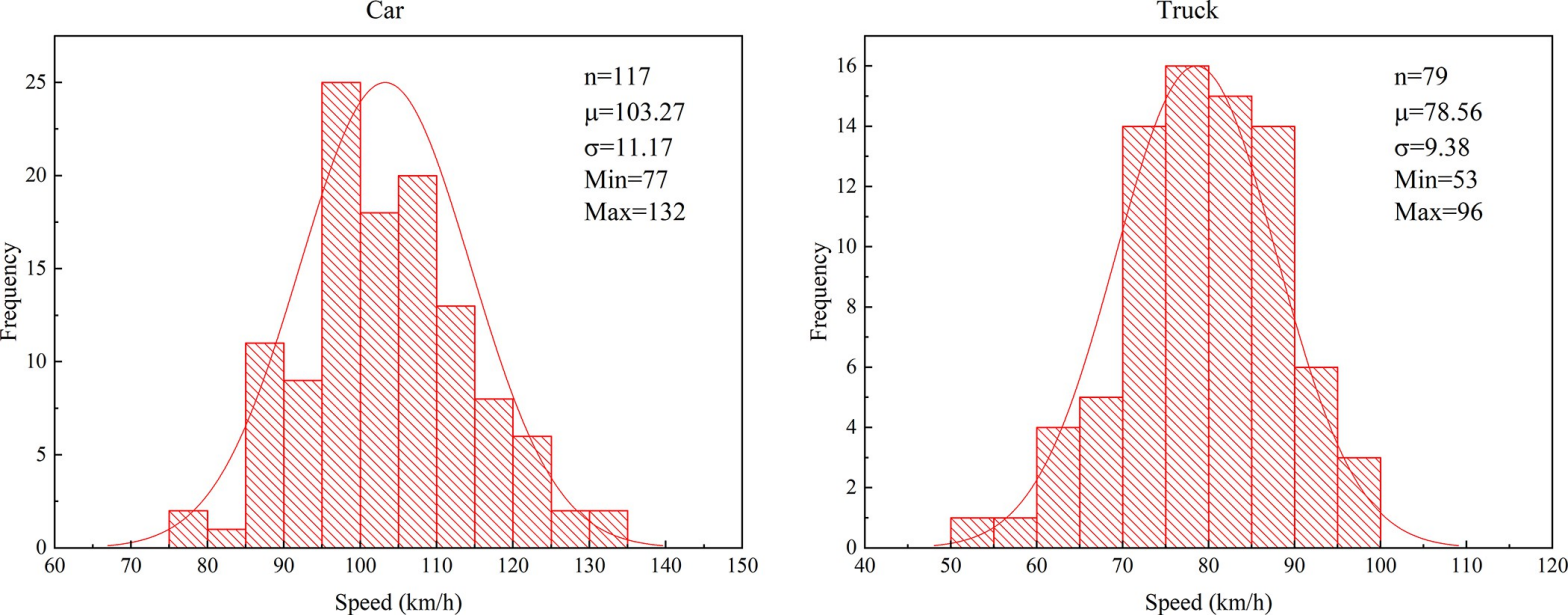
Speed information: (a) Car, (b) Truck.

Laser guns are used to collect speed data of cars and trucks in free flow conditions. During the data collection process, the experimenters concealed themselves in the bushes beside the motorway to prevent the drivers from being disturbed. 117 samples of cars and 79 of trucks were effectively collected in the experiment. The specific information of the data is shown in Fig 4. The Kolmogorov-Smirnov test results of the speed data are shown in Table 2. The two-tailed asymptotic significance value is 0.2, which exceeds 0.05. Therefore, it could be considered that the speeds of cars and trucks follow a normal distribution. This finding is consistent with the research results of Sil, Nama [28] and Himes [29].

**Table 2:** Kolmogorov-Smirnov test.

Vehicles	Sample size	Normal parameters		Most Extreme Differences			Kolmogorov-Smirnov Z	Asymp.sig. (2-tailed)
		Mean	Std.Deviation	Absolute	Positive	Negative		
Car	117	103.27	11.17	0.06	0.06	-0.04	0.06	0.20
Truck	79	78.56	9.38	0.09	0.09	-0.07	0.09	0.20

#### 2.3.2 Mean profile depth (MPD)

As an important parameter to characterize the texture of the road surface, MPD has been proved by previous studies to have a normal distribution [13]. In the research of Plati and Pomoni [14], an 11-year field data collected from an urban highway in Southern Europe was utilized to investigate a long time effect of traffic on macrotexture evolution. The detailed traffic and road geometric data with regard to MPD are shown in Table 3. The mean and variance of the MPD in the initial stage are 1.26mm and 1.36mm, respectively, while the most recent ones are 0.17mm and 0.24mm. The change in mean and variance is relatively small. Therefore, this study assumes that the MPD of the road has a normal with a mean value of 1.3 mm and a standard deviation of 0.2 mm, namely MPD~*N*(1.3,0.2^2^).

**Table 3:** Traffic, geometry and MPD data on road sections [14].

Section	Geometrical design	CTV (veh)	Initial MPD (mm)	Recent MPD (mm)
A1	Straight/relatively straight alignment and slope less than 3%	22×10^7^	1.02	1.04
A2		22×10^7^	1.02	1.04
A3		22×10^7^	1.08	1.15
A4		21.7×10^7^	1.16	1.22
A5		34.2×10^7^	1.1	1.14
A6		33.6×10^7^	1.17	1.16
B1	Straight/relatively straight alignment and slope less than 3%	9.16×10^7^	1.26	1.31
B2		5.31×10^7^	1.26	1.43
B3		9.47×10^7^	1.42	1.66
B4		5.07×10^7^	1.27	1.61
B5		3.58×10^7^	1.19	1.26
C1	Higher curvature and maximum slope 3–6%	17×10^7^	1.59	1.69
C2		18.5×10^7^	1.55	1.73
C3		8×10^7^	1.34	1.5
C4		8.41×10^7^	1.27	1.46

Note: CTV-cumulative traffic volume.

#### 2.3.3 Vehicle parameters

The present study referred values recommended by Gillespie [15] in 《Fundamentals of vehicle dynamics》. For a typical car: *r*_Φ_≈0.1rad/*g*, *h*_*r*_/*h*_*g*_≈0.5, *t*/2*h*_*g*_≈1; For a typical truck: *r*_Φ_≈0.05rad/*g*, *h*_*r*_/*h*_*g*_≈0.25, *t*/2*h*_*g*_≈0.31.

## 3. Result and discussion

In this paper, Matlab is used to conduct Hasofer-Lind Method to solve the reliability and the probability of skidding. The research results will be divided into the following four parts for discussion: The first part explores the influence of different cumulative traffic volume (CTV) and annual average daily traffic of trucks (AADTT) on the friction coefficient of the road surface. In the seond part, the skidding risk of the suspension system model and the mass point model is compared. The third part evaluates the changes in skidding risk of cars with the evolution of friction coefficient caused by CTV. Furthermore, the influence of AADTT on the risk probability of cars is analyzed in the fourth part.

### 3.1 Evolution of friction coefficient

**Fig 5:**
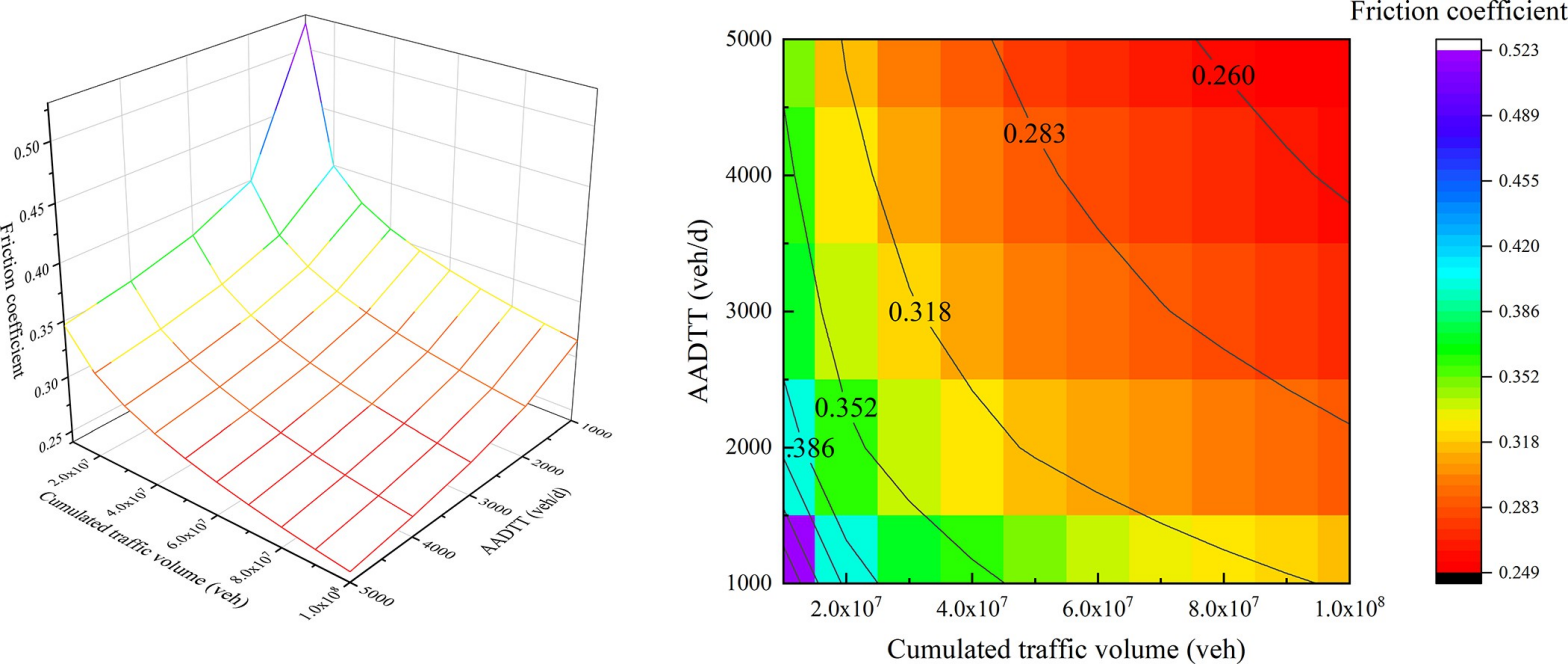
Evolution of friction coefficient under traffic flow: (a) 3D surface graph, (b) heat map.

Fig 5A and 5B show the effects of CTV and AADTT on the friction coefficient. It can be seen from Fig 5A and 5B that the friction coefficient decreases with the increase of CTV and AADTT. Furthermore, Fig 5B shows that when the CTV on the road is relatively small (CTV<20 million vehicles), the contour lines are denser, and the contour lines will be sparser with CTV increasing. It means the decrease rate of the friction coefficient is relatively swift in the initial stage of road operation, and slows down with the increase of CTV. This evolution trend of friction coefficient under the traffic polishing is consistent with that of Kane, Zhao [8].

**Fig 6:**
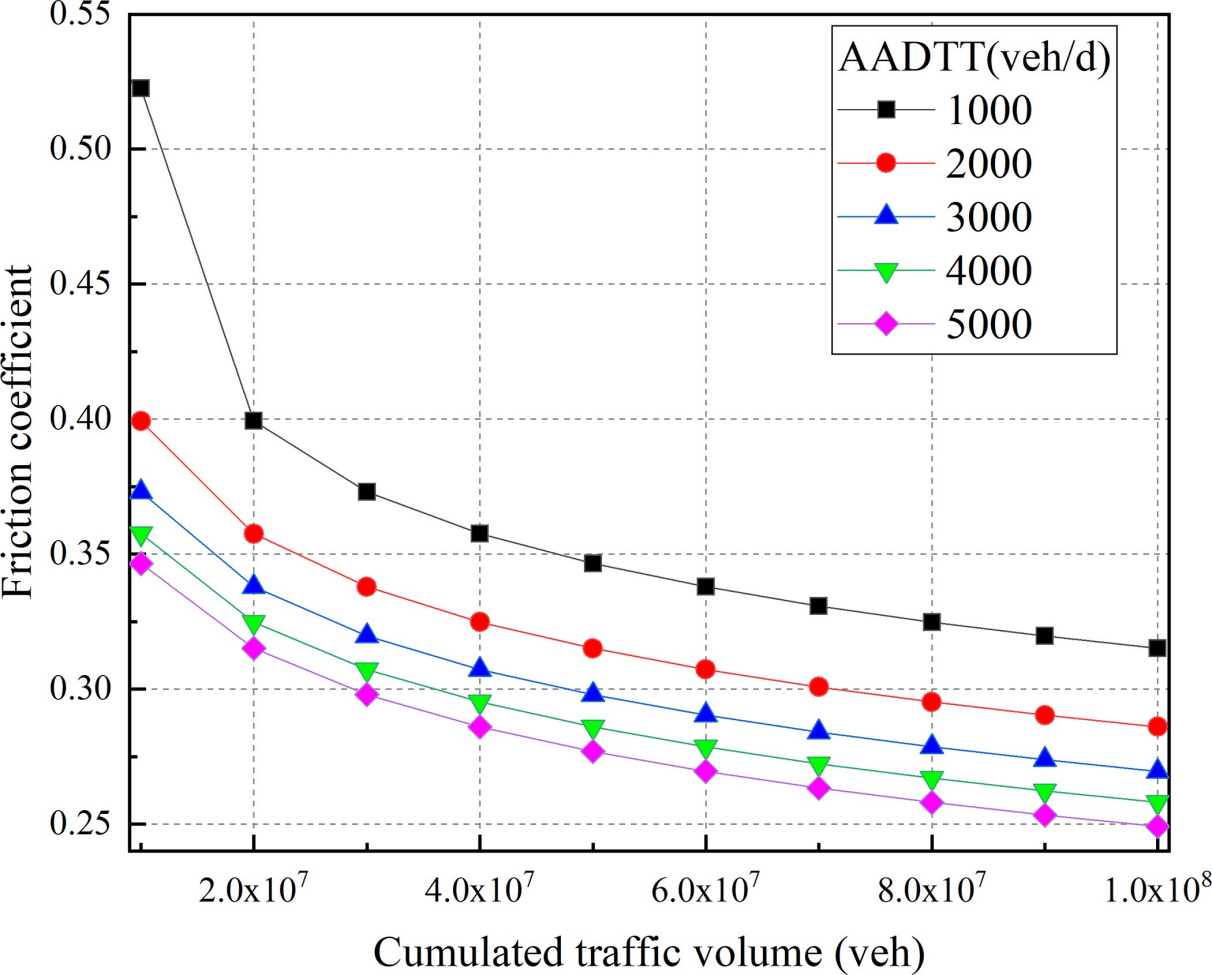
Influence of AADTT on friction coefficient.

Fig 6 shows the influence difference of different AADTT on the friction coefficient during different operating stages of the road. The difference in the friction coefficient caused by different AADTT is prominent in the early stage. As the CTV increases, the difference gradually becomes smaller. But the difference is still greater than 20%. Moreover, the larger AADTT often leads to the lower friction coefficient for the same CTV. The results of the study verify the point made by the previous study: the pavement friction coefficient is significantly affected by truck traffic volume [1, 8]. Therefore, motorways with heavy AADTT need more road maintenance works to ensure that the road can provide sufficient friction coefficient for vehicles.

### 3.2 Model comparison

**Fig 7:**
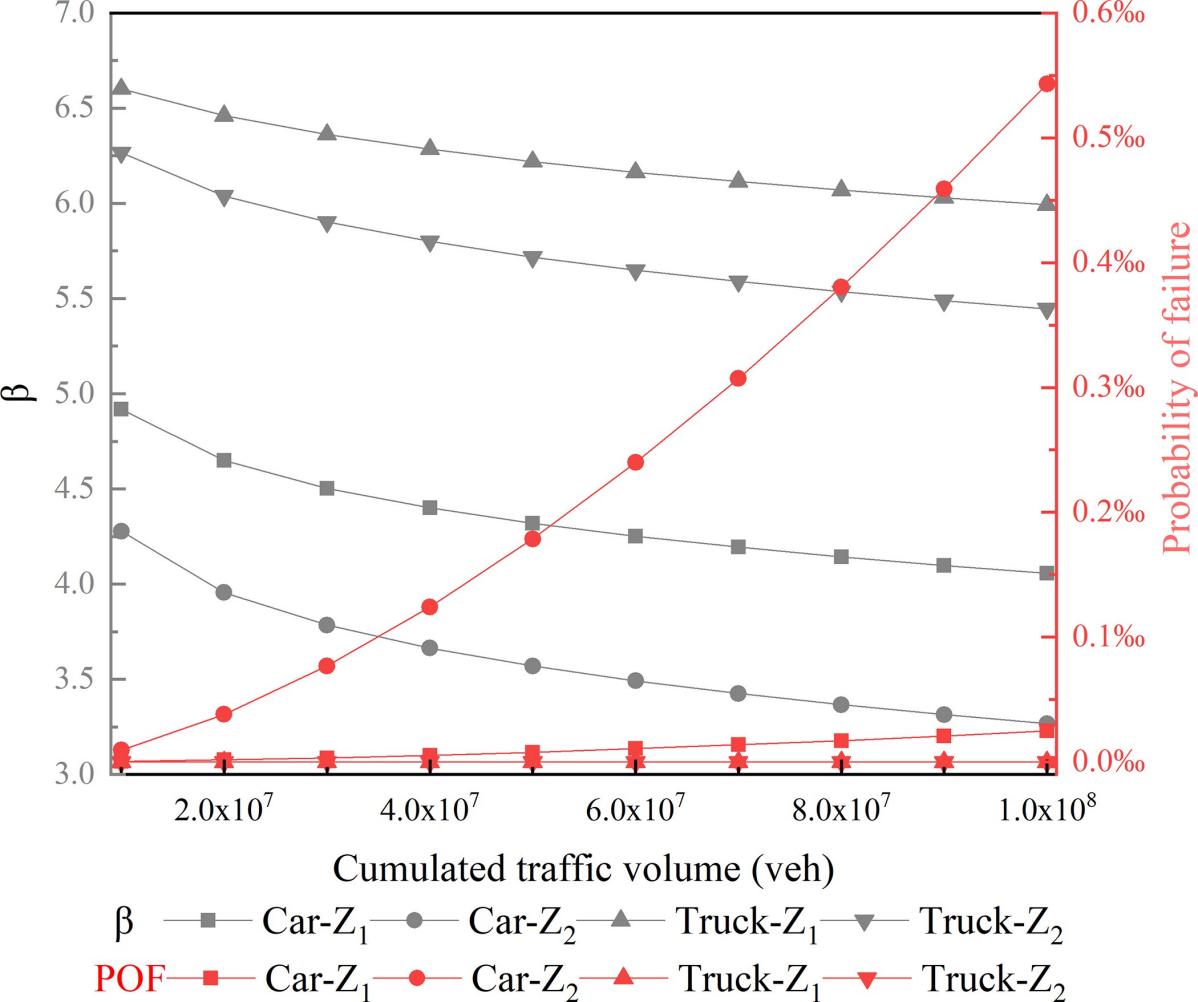
Risk comparison of different models (AADTT = 3000).

The mass point model is utilized to calculate the radius of a horizontal curve in modern road design theory [12, 23]. Some previous studies used a suspension vehicle model to evaluate the risk for the cars driving on a horizontal curve [18, 30]. You, Sun [31] found that the vehicle’s suspension system significantly affected the safety of the vehicle. Fig 7 compares the reliability and the skidding probability of failure for the mass point model Z_1_ and the suspension vehicle model Z_2_ under different CTV with AADTT of 3000 veh/d. It can be found from Fig 7 that the reliability of Model Z_2_ is lower than that of Model Z_1_ for both cars and trucks, which indicates the vehicle’s suspension system reduces the safety on turning. This conclusion is consistent with that of You, Sun [31].

Nevertheless, it is worth noting that for trucks, the probability of failure of the model with the suspension system and the mass point model is extremely little (close to 0) compared with the cars. The reason may be that the driving speed of trucks is so low. However, although the suspension system also has less impact on the safety of the cars for a small number of the CTV, the difference between the probability of failure of the model Car-Z_2_ and Car-Z_1_ will become more and more significant with the operation time going. It means that the mass point model adopted by current road design theory underestimates the risk of cars. Furthermore, the potential dangers caused by this underestimation will be magnified over time. This model evaluated the risk difference between the mass point model and the suspension car model from the perspective of road operating time, which could help road designers recognize the influence of suspension system for vehicle safety.

### 3.3 The influence of CTV on skidding risk

**Fig 8:**
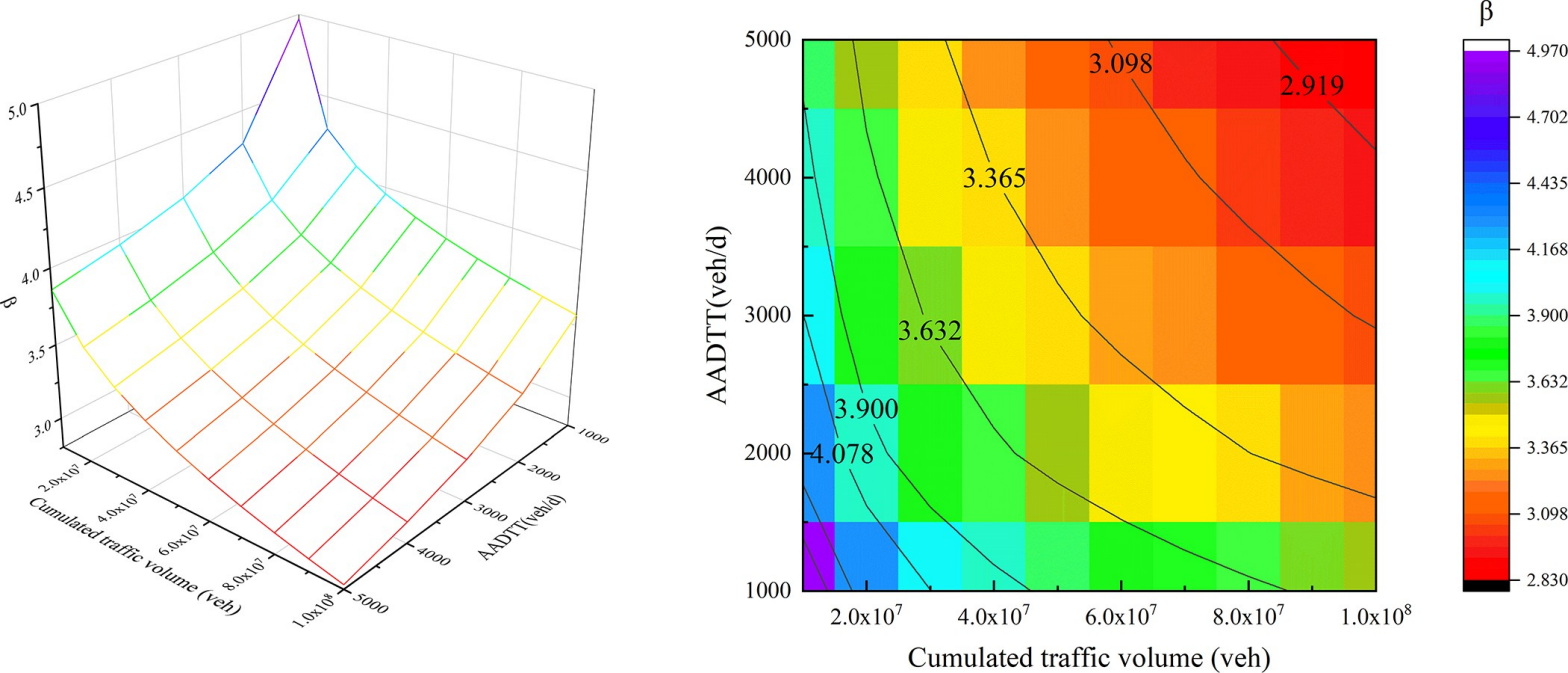
Reliability changes under the action of traffic flow (Car-Z2): (a) 3D surface graph, (b) heat map.

From the above analysis, it can be concluded that the skidding probability of cars with a suspension system on a horizontal curve is much higher than that of trucks. Therefore, the risk assessment of the suspension car model will be carried out below. The reliability changes with different combinations of AADTT and CTV are shown in Fig 8A and 8B. The change in reliability is similar to that of the friction coefficient. When the CTV is small, the reliability decreases swifter. By contrast, the decrease speed becomes slower as the CTV increases. Besides, when AADTT and CTV exceed 3000 veh/d and 60 million vehicles respectively, the reliability starts to approach 3, which shows that the reliability will involve in pauta criterion. In other words, the skidding probability will reach to 99.73% for the cars traversing the horizontal curve.

**Fig 9:**
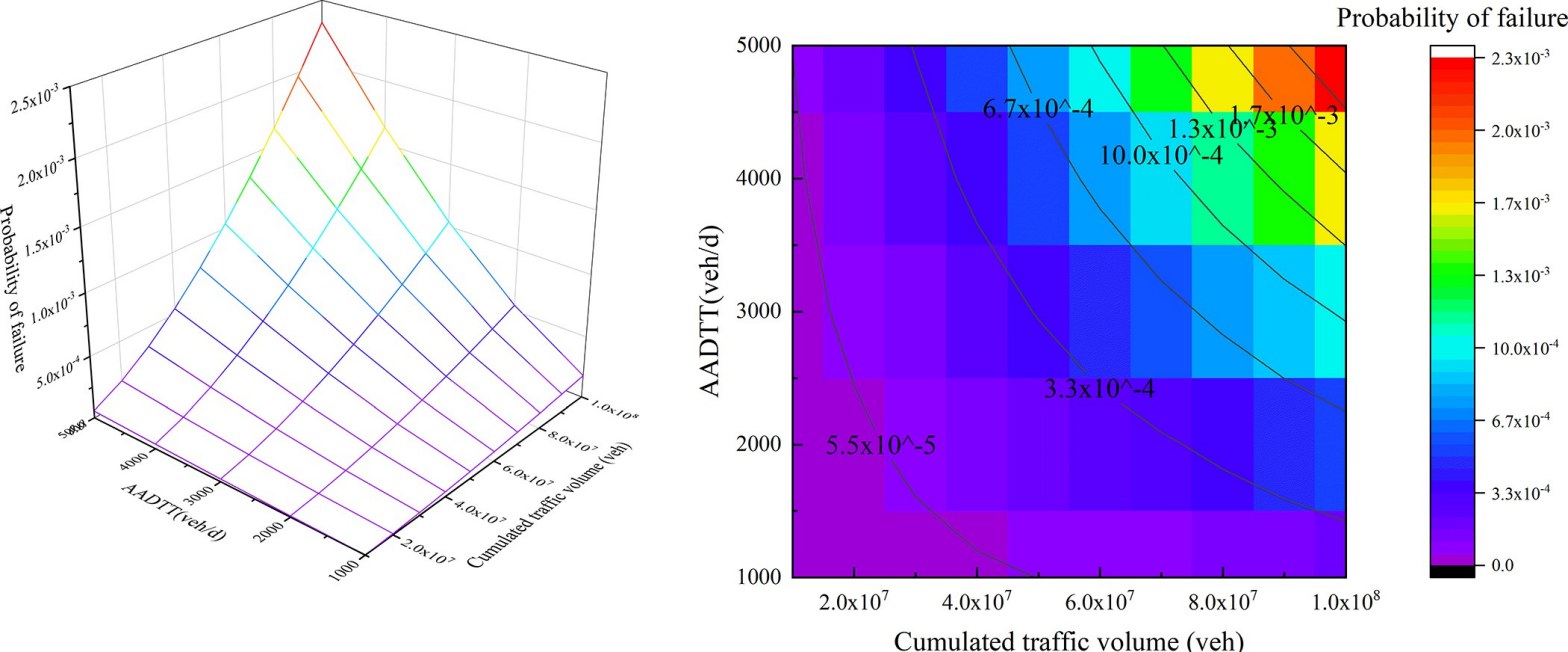
Changes in the probability of failure under the action of traffic flow (Car-Z2): (a) 3D surface graph, (b) heat map.

Although the reliability declining speed slows down with the CTV increasing, it does not the same mean so for the road safety. Fig 9 shows the change of the skidding probability for a car. This changing trend is opposite to the reliability. The probability of failure increases slowly under the low CTV. But when the CTV exceeds 50 million vehicles, the risk of the horizontal curve will increase sharply with CTV growing. This result can help road management departments recognize the influence of friction evolution caused by cumulative traffic on traffic safety, and formulate appropriate road maintenance strategies on a regular basis.

### 3.4 Risk assessment under different AADTT

**Fig 10:**
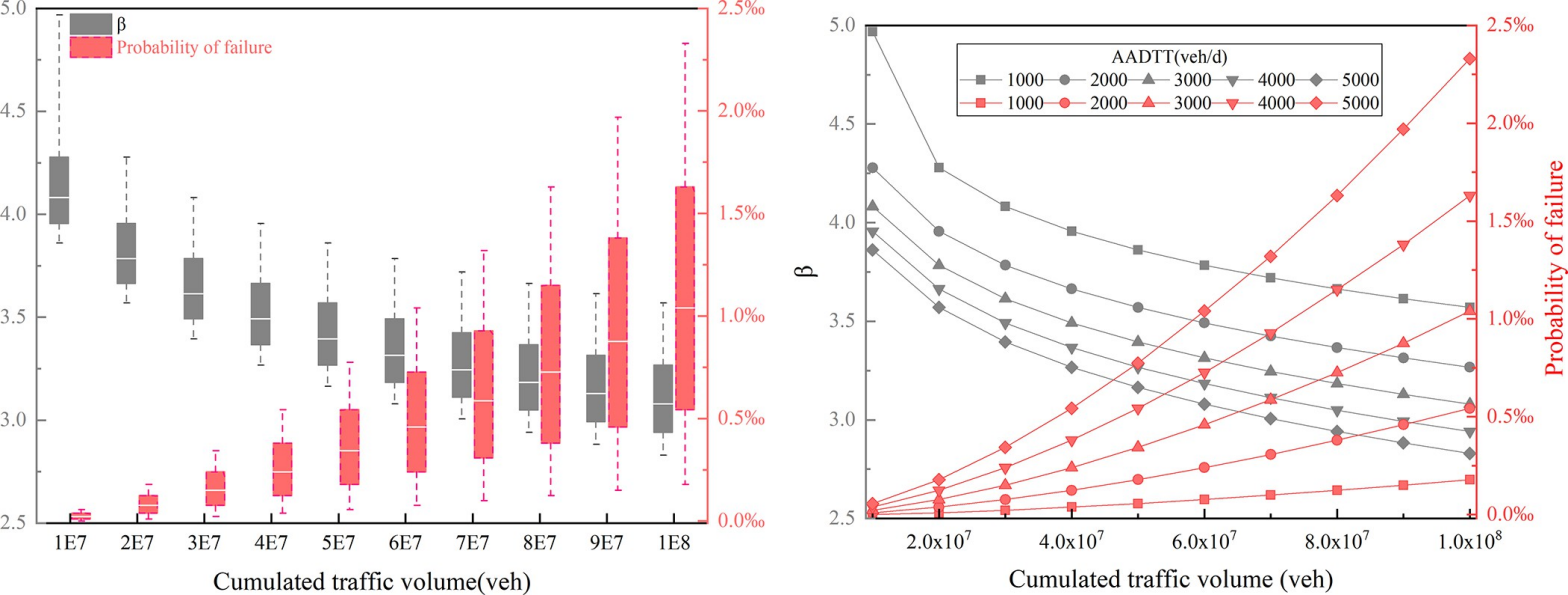
Influence of AADTT on risk (Car-Z2): (a) Difference in the influence of AADTT on reliability and probability of failure, (b) the influence of AADTT on reliability and probability of failure.

Fig 10A depicts the difference in the influence of AADTT on reliability and probability of failure. In the different operation stages of the road, the reliability difference caused by AADTT remains stable. However, the difference of failure probability becomes more and more significant with the CTV growing.

The influence of AADTT on reliability and probability of failure is shown in Fig 10B. When AADTT is less than 2000 veh/d, CTV has less influence on the probability of skidding. The growing rate of failure probability increases with the increase of AADTT. This finding shows that AADTT not only critically affects the probability of skidding, but also affects the speed of the skidding probability increase. The results mean that for roads with heavy AADTT, timely road maintenance work can not only reduce the skidding risk of cars, but also slow down the speed of risk increase.

## 4. Conclusions

Based on the reliability theory, this paper proposes an innovative risk assessment model to evaluate the influence of the evolution of the friction coefficient caused by cumulative traffic volume on traffic safety. The relationship between the friction degradation and two traffic flow characteristics, CTV and AADTT, is quantified. Then probability of failure (POF) and reliability are employed to appraise the effects of this friction degradation on the skidding risk. The main conclusions from this study are summarized as follows:

1. The polishing from CTV has considerable influence on pavement friction. The friction decrease ratio is relatively faster in the initial stage of road operation and slows down with the increase of CTV. In addition, it is found that AADTT has a considerable impact on friction evolution. Possible reasons might be that truck tires make a strong polishing effect on road surface.
2. The pavement friction reduction due to CTV has little influence on the skidding risk for point-mass model compared with the suspended vehicle model. Instead, risk of skidding will rise more quickly with friction reduction for suspended vehicle model. Furthermore, it is worth noting that the risk difference between two models is magnified as the road operating time goes.
3. As the CTV increases, friction decrease due to traffic polishing will result in higher skidding risk on the horizontal curve and swifter rising speed of skidding risk. In addition, we found that the truck traffic volume (AADTT) also has a positive effect on road risks and the speed of risk increase. The research conclusion shows that it is necessary to carry out road managements and maintenance works on a regular basis. On the other hand, this conclusion also means that for motorway with high traffic volume of truck, it is necessary to pay attention to road risk changes in time.

Based on the reliability theory, this paper evaluates the vehicle’s skidding probability with friction evolution due to traffic. The described approach and findings need to be confirmed through more experimental work, where other factors (e.g. lane width, lighting condition) not included in this analysis can be investigated further.

## Supporting information

S1 Dataset (XLSX)
